# Immunomodulation by Processed Animal Feed: The Role of Maillard Reaction Products and Advanced Glycation End-Products (AGEs)

**DOI:** 10.3389/fimmu.2018.02088

**Published:** 2018-09-13

**Authors:** Malgorzata Teodorowicz, Wouter H. Hendriks, Harry J. Wichers, Huub F. J. Savelkoul

**Affiliations:** ^1^Cell Biology and Immunology Group, Department of Animal Sciences, Wageningen University & Research, Wageningen, Netherlands; ^2^Animal Nutrition Group, Department of Animal Sciences, Wageningen University & Research, Wageningen, Netherlands; ^3^Department of Nutrition, Faculty of Veterinary Sciences, Utrecht University, Utrecht, Netherlands; ^4^Food and Biobased Research, Wageningen University & Research, Wageningen, Netherlands

**Keywords:** Maillard products, advanced glycation end products, RAGE, animal feed, immunity

## Abstract

The immune system provides host protection to infection with pathogenic organisms, while at the same time providing tolerance upon exposure to harmless antigens. Thus, an impaired immune function is associated with increased susceptibility to infections with increased disease severity and thereby necessitating the therapeutic use of antibiotics. Livestock performance and feed efficiency, in addition to their health status, are dependent on the microbial load of their gut, the barrier function of the intestinal epithelium and the activity of the mucosal immune system, all of which can be modulated by dietary components. The majority of feeds that are consumed in pets and livestock have been processed. Processing promotes a non-enzymatic reaction between proteins and sugars called Maillard reaction (MR). Maillard reaction products (MRPs) and advanced Maillard reaction products (AGEs) determine taste, smell, and color of many food products therefore the MR is highly relevant for the feed industry. MRPs interact with different types of immune receptors, including the receptor for advanced glycation end products (RAGE) and immunomodulatory potential of feed proteins can be modified by Maillard reaction. This MR has become an important concern since MRPs/AGEs have been shown to contribute to increasing prevalence of diet-related chronic inflammatory states in the gut with negative health consequences and performance. The immunomodulatory effects of dietary MRPs and AGEs in livestock and pet animals are far less well-described, but widely considered to be similar to the relevant concepts and mechanisms obtained in the human field. This review will highlight immunological mechanisms underlying initiation of the innate and adaptive immune responses by MRPs/AGEs present in animal feeds, which are currently not completely understood. Bridging this knowledge gap, and taking advantage of progress in the human field, will significantly improve nutritional quality of feed and increase the prevention of diet-mediated inflammation in animals.

## Introduction

### Protein quality of animal feeds

Dietary proteins are a source of amino acids and the ability to absorb amino acids and use them for protein synthesis determines their quality and their required dose to meet the requirement (1). The efficiency with which individual amino acids are utilized for e.g., development, growth, immunocompetence, and lactation depends not only on the concentration of amino acids and their bioavailability in foods, but also on the relative proportions to each other. Protein quality and synthesis decrease when the amino acids in the food are imbalanced or even absent, e.g., tryptophan is the least abundant amino acid in foods, while lysine is often the limiting essential amino acid in animal feeds (2, 3). Proteins are essential in foods, not only for its nutritional value, but they also determine food structure, perception, and immunomodulatory capacity. These functional characteristics are dependent on physico-chemical conditions like pH, ionic strength, temperature, or pressure, and the individual behavior of protein and their amino acids are largely unknown and unpredictable. There is a need to better understand these parameters in food production as they, have a significant effect on food quality. Often enzymes are part of these dietary proteins and these include proteases, hydrolyzing large protein molecules into smaller peptides, and peptidases, releasing single amino acids from terminal ends of proteins and peptides. Mainly proteases are used in processing to improve visual appeal, taste, yield, nutritional value, and physical properties of dietary proteins. Processing technologies affect the quality of protein foods and thereby also its safety and for this reason novel processing technologies, e.g., high pressure processing, pulsed electric field, radiofrequency and cold plasma, have been developed.

Amino acids contained within the dietary protein can undergo crosslinking and glycation reactions, including the “Maillard reaction” (MR) or glycation, which is named after the French physician and chemist, Louis Camile Maillard, who in 1912 described this reaction for the first time (4). It was, however, John Hobbs in 1953 who proposed the mechanism for the chemistry of the Maillard reaction as know it today (5). The MR is also known as the non-enzymatic browning reaction and typically involves amino acids (e.g., lysine, arginine) and reducing sugars (e.g., fructose, glucose) that progresses via a series of chemical rearrangements resulting in the formation of MR products (MRPs). MRPs include Schiff bases, Amadori products and consequently advanced glycation end products (AGEs) as the final products. The advanced products are produced via either an oxidative or non-oxidative pathway. Pentosidine and N-(carboxymethyl)lysine (CML) are examples of glycoxidation (glycation and oxidation) and pyrraline is a product of a non-oxidative pathway (6). The MR is positively correlated with the temperature and the duration of heating of proteins in the presence of reducing compounds predominantly sugars. Higher temperatures increase the reactions where long-term thermal processing with relatively lower temperatures may result in comparable results. The type and concentration of available sugars in the environment are linked to the progression of the reaction with, for instance, glucose-rich products showing a slower glycation rate in comparison with those rich in fructose (6). Of the 20 amino acids naturally occurring in food proteins, lysine due to its ε-amino acids and arginine because of its guanidine side group are the most susceptible amino acids but also histidine and tryptophan can be involved in the MR as well as the α-amino or N-terminal amino group of any amino acid or peptide, respectively (7).

As lysine is the most reactive amino acid, modification produces modified lysine derivatives (8), including formation of a Schiff's base, which is a reversible but unstable compound. Subsequently, this base rearranges into the Amadori compound, ε-N-deoxyketosyllysine (9). The latter reaction is irreversible and can proceed further into advanced Maillard reaction products, and ultimately give rise to melanoidins, leading to brown coloring of processed feeds. Because amino groups are involved in several steps of the Maillard reaction, a strong decrease of the availability of amino acids can occur. Amadori products and AGEs resulting from the MR may absorbed, metabolized by gastrointestinal bacteria to other components or leave unaltered via the feces. Once absorbed AGEs may be metabolized to other components, retained in the body or leave unaltered via the urine. Melanoidins are only partially digested and absorbed by the intestines and may be retained in the kidneys. In contrast to high molecular weight melanoidins, low molecular weight non-absorbed melanoidins are degraded in the intestines (10).

### Food processing

Food processing has been a routine procedure for increasing the taste, safety, texture, longevity, and bioavailability of nutritional components (11). Humans have long been utilizing different methods such as salting, fermenting, smoke processing, and heating in order to get to conserve food and obtain desirable product features. Among all the above-mentioned procedures, thermal processing is the most commonly used method of our modern time. Ever since the exploration of fire, cooking raw materials improved the taste and digestibility of food. High temperatures have been widely used by the food industry with a number of these processes involving product temperature reaches up to 250°C to changes the appearance and occasionally the structure of the food. Food processing may result in products of similar composition, but with markedly different physiological effects, due to differences in structure and physicochemical properties. In general, food processing has a major influence on its nutritional components and modifies biological properties of biomolecules resulting in various effects on consumer's body once ingested (2, 12). Some of these modifications have a direct and instant effect on the user such as improved olfaction and gustation as well as digestibility of dietary components (e.g., starch, amino acids). Other modifications may show their influence even years after processed foods have been consumed. The latter is mainly the results of the accumulation of modified components in the body and their gradual effects on cells and tissues over time. The accumulation of AGE-modified proteins correlates with the pathogenesis of several chronic inflammatory diseases including diabetes, rheumatoid arthritis, and Alzheimer's disease (13–17). The focus of this review is on immunomodulatory effects of dietary MRPs and AGEs in livestock and pets as this is far less well-known than in the human situation and have therefore used relevant knowledge from the human field. Immunomodulatory activity of MRPs and AGEs in feeds are based on concepts and mechanisms that are equally relevant and applicable in humans and animals.

### Advanced glycation end products (AGEs)

The final glycated products of the MR are commonly referred to as glycation end-products (AGEs). In laboratory animals and humans, consumption of large quantities of such AGEs were shown to induce pathological alterations and therefore AGEs are increasingly subject of safety studies (18). Advanced glycation end products are either produced endogenously (*in vivo*) due to physiological processes such as oxidative stress and aging or are formed exogenously (in food) following the progression of the MR (19). Glycation changes protein structures and this may result in malformation and malfunction of affected proteins. Small endogenously formed glycated and misfolded proteins are targets for intracellular degradation by the 20S proteasome of the Ubiquitin-proteasome-system (UPS) (20). However, these small proteins when oxidized further and following cross-linking reactions, may form larger structures. The formation of big and bulky glycated proteins blocks the activity of proteasomes and makes them resistant to degradation leading to the accumulation of such molecules in the cells and tissues (13). Another mechanism for eliminating AGE-modified proteins is via the lysosomal system. Cellular receptors recognize these modified proteins (especially from exogenous sources) and internalize them into endosomes. The AGE-containing endosomes are then fused to cytosolic lysosomes where lysosomal proteases process and break down these modified proteins (21). The degraded peptides are then cleared from the body through the urinary system with a, hitherto, poorly defined mechanism.

## Advanced maillard reaction products and their possible effects on health

### MR and damaged dietary proteins

The most common form of dietary AGEs are protein-bound, while some are either in free state of bound to peptides. These protein-AGEs are enzymatically hydrolyzed into small fractions that can either be absorbed in the small intestine of remain unable to be absorbed, and thus is absorption related to digestibility of the dietary AGEs (22). The abundance of α-dicarbonyl groups in processed feed and their relevance in the formation of AGEs, determines the digestibility, nutritional value and health impact of protein-AGEs (23).

The MR can occur at temperatures similar to that of the human body (24). As an example, a fraction of hemoglobin (HbA0) reacts with glucose under *in vitro* conditions, yielding the MR product HbA1c and further reaction products including Schiff's base and Amadori compounds. In diabetics high concentrations of HbA1c are present and these consist of α-amino-1-deoxyfructose at the N-terminal valine amino acid in the β-chain (24).

The concentration, nutritional value and digestibility of amino acids (esp. lysine) in feed ingredients and diets may be reduced due to heat treatment of feed ingredients (10). Feeding broiler chicken or weanling piglets a heat-damaged soybean meal diet, a decrease in body weight and carcass weight was observed compared to feeding untreated soybean meal. These negative effects of heat damage on performance, however, were partially mitigated by adding crystalline amino acids to the diets (25). Heat damage also may cause losses in vitamins, e.g., loss of vitamin B6 and thiamine when storing milk powder at 70°C (26).

The structural and functional properties of proteins can be modified due to covalent interactions and the cross-linkage of proteins during the formation of such AGEs. The resulting resistance to digestion, delays the turnover rate of these proteins and this accumulation of AGEs may hinder tissue repair (27). Moreover, these Ages bind to receptors widely expressed on tissue cells and as a consequence oxidative stress, vasoconstriction, excessive collagen deposition, and inflammatory responses are stimulated (27–30). The development of chronic systemic inflammation (metaflammation) can be the consequence of prolonged exposure to AGEs and metaflammation is observed in many cancers in both humans and dogs (31). Canine diets should therefore limit stimulation of the AGE/metaflammation axis resulting in less carcinogenic activity. Such diets offer opportunities to be tested for AGE and metaflammation accumulation that result in lower prevalence and incidence of cancer in dogs.

A low MRP diet in a mouse model resulted in decreased body weight, lowering of insulin concentration during fasting, increased HDL levels in plasma, and reduction of a high-fat diet-induced insulin resistance (32). Also in human diabetics, complications associated with impaired wound healing were improved when MRPs were avoided in the diet (3, 32).

Advanced glycation end products have been associated with the etiology of age-related diseases in humans, such as atherosclerosis, nephropathy, retinopathy, osteoarthritis, neurodegenerative diseases, and diabetes mellitus. Also in dogs such age-related diseases occur, with many similarities to humans (33). In aging dogs with e.g., diabetes mellitus, increased tissue levels of AGEs were found (34), but also in conditions like carteracts, osteoarthritis (35), neurodegenerative canine cognitive dysfunction syndrome (28), vascular dysfunction, and atherosclerosis (29, 30). The limited exposure to dietary AGEs and a reduced AGEs pool during calorie restriction could explain the widely described beneficial effects on aging and related complications (36–40).

### Adverse effects of MR on bioavailability of lysine and other dietary compounds

The MR importantly results in blockage of lysine thereby reducing the biological availability of the lysine amino acid but also together with the crosslinking hinder hydrolysis of the protein by digestive enzymes. This has has been demonstrated in animal feed ingredients, like dairy products (41), dried grains used as the feed for pigs, soybeans (42), carrots (43), peas (44), and maize (45). The thermally induced reduction in lysine bioavailability in the presence of sugar, depends on the level and duration of temperature application, water activity and pH of the environment during processing (46). Furosine (ε-N-(furoylmethyl)-L-lysine), which is related to the early stage of the MR, is an indicator of the formation and presence of Maillard products such as fructoselysine, lactuloselysine, and lysinoalanine and of the losses in available lysine.

Next to the impaired bioavailability of lysine, MRPs have a strong mineral chelating power affecting the availability of minerals such as calcium, iron, and phosphorus (47, 48).

Rérat et al.(49) used an *in vivo* pig model to study feeding of a protein source with a high level of MR-induced blocked lysine on the kinetics of digestion, nutrient absorption and excretion, including amino acids but also minerals. The pigs were fed with non-processed milk (MR free) or skimmed milk processed to obtain about 50% of lysine blockage due to the early stages of Maillard reaction. Consumption of processed skimmed milk induced a lower absorption of milk sugars glucose and galactose. This was in part due to the loss of milk-derived lactose as this was converted into lactuloselysine and lactulose. In addition, reduced amounts of lysine, cysteine, and alanine were found due to absorption and these appeared in the portal blood in pigs fed the processed skimmed milk suggesting that lactuloselysine was not bioavailable. The fecal excretion of amino acids was higher in the group of pigs fed processed skimmed milk confirming the impaired digestibility of proteins modified via MR (49). Thermal processing of milk was shown to induce damage of casein resulting in a decreased bioavailability of lysine and this lysine blockage was reflected in the lower growth rate of kittens fed with heated casein (50). A decreased protein digestibility results in a diet high in the MRPs and consumption led to 47% higher excretion of fecal nitrogen, 12% lower absorption of nitrogen, and a 6% lower nitrogen digestibility in a group of adolescent males (51). The MRP-rich diet was also shown to affect the absorption of phosphorus, resulting in a decrease of the phosphorus balance (52). Some MRPs can directly inhibit brush border enzymes as shown for glucose-lysine reaction compound (2-formyl-5-(hydroxymethyl)pyrrole-1-norleucine) *in vitro* but also *in vivo* in a rat study (53, 54).

Moreover, fructoselysine, an Amadori adduct of glucose to lysine, was detected in the portal blood and urine of pigs fed with processed skimmed milk in amounts corresponding to 18.6% of the ingested quantity. This suggested that the galactose residue of lactuloselysine is released by enzymes in the gut lumen and/or in the epithelial brush border and subsequently transported through the intestinal barrier (49). Both early and advanced glycation end products were detected in the blood of rats after feeding a MR-rich diet. Approximately 26.0 to 29.0% of ingested dietary CML in rats was excreted in urine, compared to 15.0 to 22.0% in feces (55). In humans, protein-bound fructolysine urinary excretion ranged from 1.4 to 3.5% of the ingested amount (56). In contrast, about 10% of diet-derived AGEs were absorbed in healthy subjects, and two thirds of these AGEs remained in the body while one-third of the absorbed AGEs was excreted into the urine within 3 days. Low molecular weight AGEs are water-soluble and are not substrates for liver detoxifying enzymes and therefore rapidly excreted. Consumption of high molecular weight pentosidine led to excretion of only 2% AGE resulting in accumulation, endothelial perturbation and vascular disease (57).

### Influence of cross-linking immunogenicity of proteins

The MR but also heat treatment alone during thermal processing of food can result in cross-linking of the proteins. Methylglyoxal, a common intermediate in the MR *in vivo* and *in vitro*, has been shown to be involved in the formation of cross-linked aggregates via lysine, arginine, or cysteine. Normal physiological concentrations of methylglyoxal are sufficient to induce these reactions resulting in different fluorescent products that resemble proteins characteristic for aging and diabetes development (58–60). Furthermore, dehydroalanine may react with lysine and cysteine residues to form cross-linked products such as lysinoalanine (LAL) and lanthionine (61). LAL was found in the urine, plasma, liver and kidneys of rats fed with heat-modified casein (55). However, the study of Hellwig and colleagues suggests that LAL is broken down during the digestion process into larger peptides (61). Next to the formation of LAL, MR, and/or denaturation of proteins during the heat treatment may initiate hydrophobic interactions between the proteins and formation of new disulfide-bonded aggregates (62). The cross-linking of proteins decreases their digestibility (63) but also affects the immunogenicity and allergenicity of proteins (64–67). Roth-Walter and colleagues demonstrated an impaired uptake of aggregated β-lactoglobulin and α-lactalbumin by intestinal epithelial cells. In a mouse model, protein aggregation was shown to increase the uptake into Peyer's patches. Compared to non-aggregated proteins, this uptake promoted significantly higher mucosal Th2-associated antibody responses and cytokine production profiles (64). Also exposure to cross-linked β-lactoglobulin was shown in mice to elicit a stronger allergic sensitization probably due to enhanced resistance to gastrointestinal proteolysis, retrogradee protein transport to Peyer's patches, and an altered uptake and processing in antigen-presenting cells (65). Liu and colleagues showed that agglomeration of whey proteins during heating is positively correlated with the decreasing water activity and progress of the MR. Moreover, the formation of aggregates was associated with the formation of ligands binding to the cell-bound but also sRAGE (the soluble form of receptor for advanced glycation end-products), which reflects increased immunoreactivity of MR-modified agglomerates (66, 67).

## Immune-related effects

### Influence of AGEs on immune system

In general, AGEs affect biological procedures in three levels; the first effect is an alteration in signal transduction pathways, which happens following the AGE-receptor interaction. Secondly, via altered signaling, AGEs induce or inhibit the production of certain cytokines, hormones, and free radicals. Finally, as a result of AGEs effects and increased pro-oxidative activities, the proteins in the target tissues modify leading to functional deregulations (68).

There is substantial evidence on the association of MRPs with immunity stimulation and immune system responses (69, 70). The interaction begins with the recognition of MRP's conformational epitopes by the pattern recognition receptors (PRRs) and subsequently a downstream signaling to the nucleus for mainly, NF-kB activation and consequent cellular responses (71). Multiple PRRs have the potential recognition ability and binding affinity for MRPs but their interaction may lead to various responses (72, 73). Both early and advanced glycation products are related to an increase in oxidative stress along with inflammatory response (70, 74). As a consequence of interactions of AGE to the RAGE receptor, increased production of pro-inflammatory cytokines such as TNF-α, IL-1β, and IL-6 occurs in endothelial cells and monocytes (75). An increase in free radicals and oxidative stress aggravates the inflammatory state (auto-amplifying) and may eventually affect long-lived proteins such as collagen and elastin (76).

MRP and AGEs that are absorbed in the gut and arrive in the mucosal tissue are being confronted with the local mucosal immune system. This exposure induces immune activation leading to local effects in the gut, including induction of tissue damage and inflammation, and the start of an activated immune response leading to T-cell activation and (IgA) antibody production. Besides a local activation of the immune system in the gut, activated immune cells can also travel to the mesenteric lymph node and arrive through the portal vein into the liver and systemic circulation, leading to consequences in the entire body. One such example of a systemically activated immune response is allergy. Dietary AGEs (dAGEs) have various allergenicity and immunogenicity properties in terms of food allergy (77, 78). The mechanism for initiation and progression of the reactions are still under debate. However, it was shown that depending on the structure and type of glycated proteins, these molecules might increase or attenuate allergic reactions. An increase in allergenicity of roasted peanut with an accompanied increase in IgE reactivity was reported (79, 80). Besides, Hilmenyuk et al. demonstrated that mature DCs loaded with AGE-modified proteins induced a higher T helper 2 (Th2) response (81) what promotes an allergic reaction. In addition, the cross-linking between the proteins as a result of heating makes them resistant to proteolysis and also may increases the allergenicity properties of the products. That was confirmed by increased sensitizing capacity of glycated β-Lactoglobulin when compared to the native form of the molecule (65), which could be because of neo-allergen generation. In contrast, heating may also denature the proteins and consequently, destroy or mask the conformational epitopes that leads to a reduced allergenicity (82).

### Cellular receptors for AGEs

A series of cell surface receptors present on antigen presenting cells (APCs) have an affinity to bind and interact with AGEs. According to the glycoprotein structure of AGEs, these receptors mostly have carbohydrate recognition domains (CRDs) and/or domains to interact with available peptides. There is a wide range of cellular receptor with such characteristics, however, not all of them bind to AGEs. In addition, the AGE receptors do not bind to modified proteins with similar affinity, which may lead to dissimilar responses. Taking into account the studies related to AGEs, six membrane-associated receptors are considered valuable.

#### RAGE

RAGE or receptor for advanced glycation end products is a multi-ligand receptor and is a member of the immunoglobulin (Ig) superfamily. This receptor is expressed by different cell types including monocytes/macrophages (83), endothelial cells (84), and dendritic cells (85). They recognize a wide range of molecules such as AGEs and amyloid-β peptides and are involved in activation, migration, and maturation of different cells. The presence of excessive amounts of AGEs or large increases in inflammatory conditions will up regulate RAGE expression and activation. Following their activation, Reactive Oxygen Species (ROS) generation and inflammatory responses are exerted which may lead to chronic inflammatory disorders if the stimulation persists. Three variants of the RAGE protein were described: full-length RAGE, N-truncated RAGE, and soluble RAGE (sRAGE) and all of these share various common core operational domains. The latter form (sRAGE), which has a molecular weight of ~46–50 kDa is secreted extracellular and contains the extracellular ligand-binding domain and regulates RAGE levels by negative feedback mechanisms (86).

#### Galectin-3

AGE-R3/Galectin-3 is a member of lectin family, which along with two other components (AGE/R1/OST-48 and AGE-R/80K-H) forms the AGE-R complex. This complex and mainly the extracellular ~32 kDa Galectin-3 subunit, was shown to bind with high affinity to AGE-BSA on macrophages (87). This multiple function receptor interacts with glycoproteins via its carbohydrate recognition domains (CRDs) and N-terminus domain. It is also a major regulator of biological processes including acute and chronic inflammation (88, 89). According to this capacity of Galectin-3 for interacting with glycoproteins such as AGEs and also its importance in immune responses.

#### SR-AI

Scavenger receptor class A1 (SR-AI) with the molecular weight of ~77 kDa (as monomer), belongs to the family of macrophage scavenger receptors (MSR) and consists of six different domains. Likewise other receptors of this family, SR-AI is mainly involved in mediating phagocytosis of microorganisms. Being generally expressed on monocytes and macrophages as well as DCs, these membrane-bound PRRs have versatile functions and have a wide range of ligands. In addition to microbial ligands, they bind to modified molecules including glycated proteins such as AGEs with high affinity and facilitate their endocytosis. They are one of the key role players of innate immunity responses and are involved in, for instance, macrophage polarization and pathogenesis of diseases such as atherosclerosis (90).

#### CD36

CD36 (SR-BIII) is a glycoprotein belonging to the scavenger receptor family and is present on macrophages. Similar to other members of this family, the 88 kDa CD36 has a large repertoire of ligands and binds to microbial ligands as well as modified self-molecules. This multi-function receptor participates in activities such as phagocytosis, antigen presentation and apoptotic cell clearance and contributes to inflammatory responses (91). Several studies showed CD36 as an AGE-binding receptor, which facilitates the cellular uptake of these glycated molecules (92).

#### DC-SIGN

DC-specific ICAM3-grabbing non-integrin (DC-SIGN; CD209) is a member of the type II transmembrane receptor family. It is present on the cell membrane of dendritic cells and mediates their adhesion process to T lymphocytes (93). DC-SIGN is abundantly expressed on DCs and contains a C-terminal lectin domain as it a member of the C-type lectin family (94). DC-SIGN binds to several ligands and exerts a variety of responses based on them. For instance, it was shown that following the DC-SIGN interaction with mannose-containing molecules, production of IL-10, IL-12, and IL-6 increased where after binding to fucose-containing ligands only IL-10 production was upregulated (95). This potential ability to bind to carbohydrates makes this receptor a candidate to bind modified glycoproteins and specifically AGEs.

#### MMR

The type I transmembrane mannose receptor (MR; CD206) is another member of the C-type lectin family with 8 carbohydrate recognition domains (CRDs) (96). With no capacity to bind galactose, MR preferentially binds to mannose and fucose and with a lower affinity to glucose (97). It is also shown that MR (CD206) acts as pattern recognition receptor (PRR) and enhances the uptake and presentation of antigens by DCs (98). This potential capacity to bind to carbohydrates and aiding DCs in antigen internalization makes this mannose receptor a candidate to bind AGEs.

### Antigen presenting cells

T-lymphocytes (T cells), as the effector cells of the adaptive immune system, are not able to recognize free antigens (99). Dendritic cells (DCs), macrophages, and B-lymphocytes have the ability to internalize antigens and present antigen-derived peptides to T cells on their major histocompatibility complex class II (MHC II) molecules. These cells are known as professional antigen-presenting cells (APCs) because displaying endogenously obtained peptides is one of the main integral parts of their function (100). DCs and macrophages (as mononuclear blood cells) are the primary immune cell types that establish the link between innate immunity and adaptive immune system. The APCs in general internalize and process complex antigens and subsequently, via different pathways, display the peptides on their MHC-binding groove. Following this presentation and based on the type and nature of the antigen, proliferation and differentiation of T lymphocytes begin and various forms of immune responses are generated. DCs mainly play a role in introducing antigens to naïve T cells where macrophages and B cells are involved in activating T cells in cell-mediated and humoral responses respectively (101). The whole process of antigen recognition, presentation, and activation is essential for a proper immune response to different perturbations and APCs play a key role in this regard.

**Figure 1:**
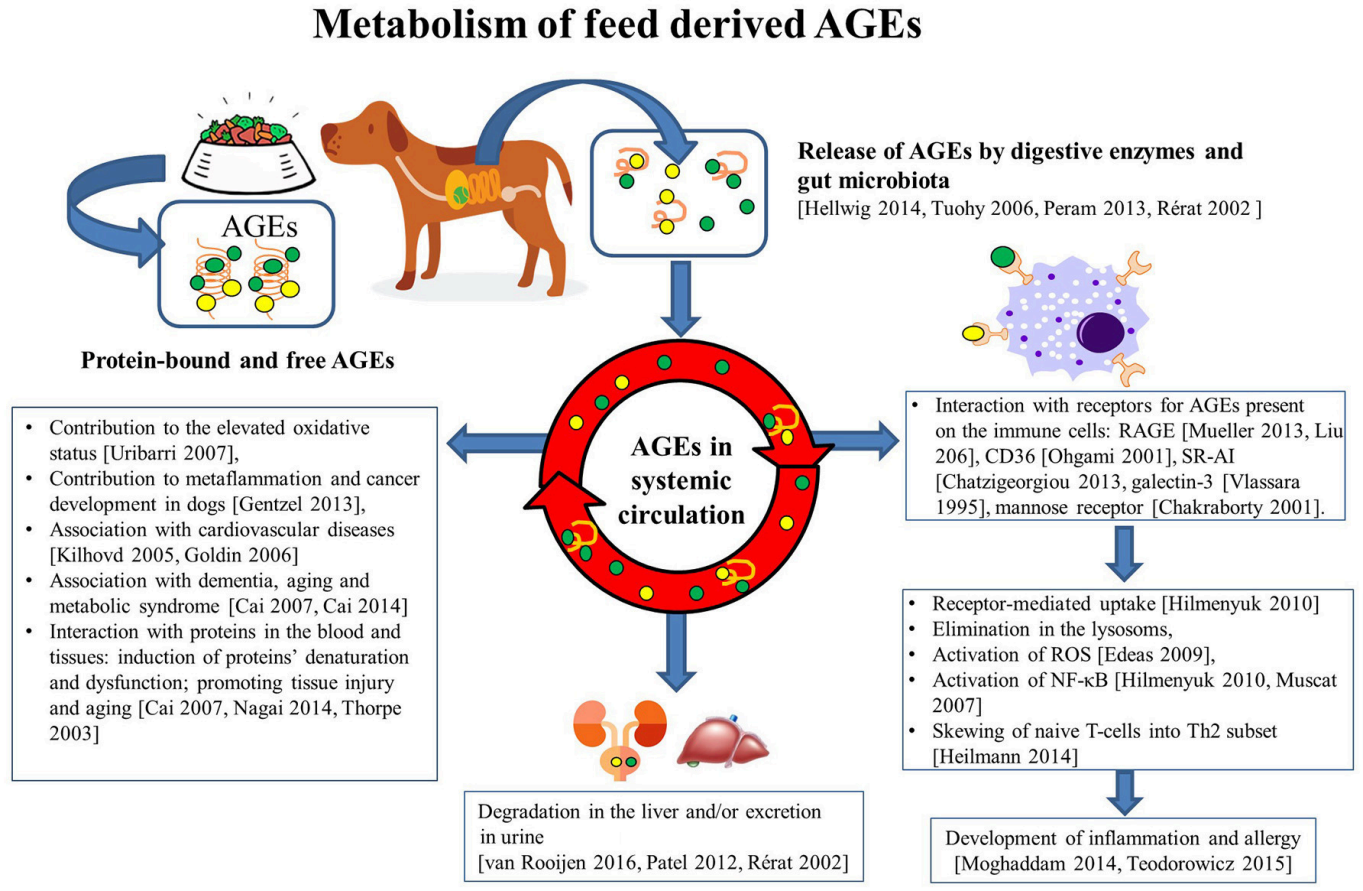
Metabolism of feed derived AGEs. Upon processing of animal feeds, protein-bound and free AGEs appear in the feed. These AGEs can be released in the intestinal tract as a consequence of the interaction with digestive enzymes and also intestinal microbiota. When absorbed, these AGE mat interact with the mucosal immune system by binding to several receptors (RAGE, CD36, Galectin-3, SR-A1, mannose receptor) expressed on immune cells. In particular, innate immune cells may subsequently become activated (through activation of the NF-kB pathway) and produce reactive oxygen species (ROS), and cytokines. Together these activities may skew the resulting T-cell activation and contribute to the development of increased oxidative stress, metaflammation and tissue damage, cancer cardiovascular diseases, metabolic syndrome and type 2 diabetes, and dementia and premature aging. The uptake of AGE in the intestinal tract can also result in appearance in the systemic circulation here these compounds will be degraded in the liver and be excreted in the urine. Collectively, these activities of AGEs explain their effects on health, welfare, growth, and performance of the animal in ways very similar to those known from the human field.

When AGEs interact with receptors expressed on APC, these AGEs can be internalized and presented in the context of MHC class II molecules to specific T-cells. In a mouse model, AGE-modified ovalbumin was phagocytosed much more efficiently by scavenger receptor class A types I and II (SR-AI/II) expressed on myeloid dendritic cells compared to non-modified native OVA. This enhanced antigen presentation led to the increased activation of ovalbumin-specific CD4+ helper T cells (81). An enhanced uptake of FITC-labeled AGE-modified ovalbumin was observed in human DCs, and this was mediated by the AGE-binding mannose receptor, scavenger receptor, and also by macro-pinocytosis by these cells. As a result, the resulting T cell activation led to an increased Th2 cytokine production (IL-5, IL-4, and IL-6), compared to the non-glycated ovalbumin-loaded DCs that induced a significant Th1 (IFN-γ) or regulatory T-cell cytokine production profile (IL-10) (102). A schematic representation of how feed derived GE affect several aspects of the metabolism are depicted in Figure 1.

## Advanced glycation end products in different animal feeds

### AGEs in milk and dried whey proteins

The dairy industry generate large volumes of liquid cheese whey that are processed to produce different whey products, including whey protein concentrate, whey protein isolate, and several individual whey proteins. These processed whey products are widely used as additives or supplements to animal feeds including sows and neonatal piglets, young ruminants (calves), dogs, cats, poultry and aquaculture (103–106). In pig industry, early weaning is becoming a common practice to boost efficient and economic production systems. The sudden change from sow's milk to solid feed and accompanied change in husbandry conditions, provide strong stressors to the piglet and its health. To minimize the impact increasingly milk replacers are being provided stabilizing gut microbiota, preventing intestinal dysfunction and improve performance (107). Cow's milk because of its balanced nutrient composition makes it a suitable feed for neonatal piglets. Even dairy cattle are fed whey proteins being a side-product of the cheese manufacturing industry. Liquid, solid, or condensed whey or products derived thereof are applied (108). Moreover, lactose and dried whey are used as supplements in poultry diets (104, 105). Lactose is not absorbed from intestine in poultry due to their inability to secrete lactase. Instead, lactose is fermented to lactic acid and volatile fatty acids (VFA), and these products promote the colonization of the intestinal tract by Lactobacilli. Elevated VFA concentrations are considered beneficial as they induce a decrease of the caecal pH and alter the oxidation and reduction potentials, and together this may suppress the growth of potentially pathogenic bacteria (104, 105). Therefore, supplementation of the diet with dried whey proteins sounds to be an effective method in enhancing the productivity of broilers.

The combination of proteins, sugar, and high temperatures during thermal processing of milk and whey proteins makes milk and other dairy products prone to glycation and creation of AGEs. In milk, whey proteins are the most heat vulnerable proteins and are subject to glycation (109). Intensive heating has been already shown to promote the formation of Maillard reaction products in whey. Intensive dry heating at a lower water activity (aw = 0.23) favors the protein aggregation and the occurrence of Maillard reaction of whey proteins (66, 67). Moreover, dry heating promoted aggregation of whey proteins and thereby the formation of sRAGE-binding ligands which influence the immunogenicity of the food compounds (66, 67). RAGE is considered an innate immunity related pattern recognition receptor that recognizes mainly conformational secondary structures, such as β-sheets and fibrils, rather than the primary amino acid sequence of proteins (110). This may suggest that thermally induced increase in the content of β-sheets also favors the aggregation of whey proteins and at the same time the binding to sRAGE (66, 67). Effects of protein denaturation during the heating are specially known for whey proteins (111). Accordingly, investigating an interaction between these AGE-modified proteins and cellular receptors on immune cells (as a crucial encounter and interaction point) seems worthwhile.

### AGEs in pet foods

Pet animals, including dogs and cats, are most often fed commercial foods that are highly processed and which they consume during their entire live. The main processing procedures for these foods rely on heat treatments (e.g., extrusion, sterilization, drying) to improve their nutrient digestibility, shelf life, and safety. As a result, the proportion of reactive lysine is on average, 73% (range 39–100%) of total lysine, while foods for growing dogs may supply less lysine content than the animals require and is recommended. As a consequence higher AGEs contents in plasma from dogs suffering from canine diabetes mellitus and impaired renal function compared with healthy control animals fed for prolonged periods with these processed foods (3).

The ingredients used to formulate pet foods and the types of processing appear to be key factors for the MRP concentrations in the final product. On average and on a dry matter basis, higher MRP and AGEs amounts occur in canned foods than in pelleted and extruded foods. van Rooijen et al. (112) calculated that the content of CML and HMF that are present in commercial pet foods are, on average, within the range reported in processed human food products. However, the average daily intake (mg/kg body weight^0.75^) of HMF was 122 times higher for dogs and 38 times higher for cats than the calculated average intake for adult humans, while the average daily intake of CML was comparable to the intake of adult humans (112). This study also highlighted the importance of measuring the reactive lysine content in foods for growing dogs used as weaning diets.

The important questions remains whether the MRP and AGEs contents reported in pet foods are physiologically relevant in these animals and this depends, in part, on the bioavailability of these MRP components. The observed increase in urinary excretion with increasing dietary intake indicates that dietary MRPs are absorbed from the gastro-intestinal tract of adult cats and excreted in the urine. The observed decrease in urinary recovery with increasing intake suggests a limiting factor in digestion, absorption, metabolism or urinary excretion. Whether such absorbed dietary MRP affect the long term health of pet animals has, hitherto, not been studied.

### AGEs in pig feed

Proteins are the main macronutrient in swine feed and, thus, understanding the absorption and the utilization by the animal is important for successful swine production and the sustainability of this sector. Storage and in particular processing conditions largely determine the nutritional value of important feed ingredients and this is most likely dependent on the combination of heating and humidity parameters that induces the formation of MRP (113). Many different processing conditions of feed ingredients are generally used in swine diets (e.g., soybean meal, dried distillers grain, corn, maize gluten feed). Heat processing to improve nutritional quality and to remove solvents that are commonly used during oil extraction is commonly required when producing oilseed ingredients (soybean meal, canola meal, sunflower meal, and cottonseed meal). These procedures comprise varying degrees of heat with the risk to be deleterious to protein quality, especially when applying high temperature regimens. As discussed above lysine, being the most important limiting amino acid in swine feed ingredients, is the most reactive amino acids in the MR. Consequently, lysine is commonly added to diets of swine in crystalline form to ensure that the balance of absorbed and available amino acids closely aligns with the requirements for protein synthesis to ensure optimal performance.

### AGEs in cattle feed

Conventionally fed cows regularly consume concentrated and processed feed containing MRP and AGEs as a consequence of heating and these compounds can be measured in the milk. Organically fed cows only get non-heated feed such as grass or silage. Thus, organic milk is supposed to contain fewer glycated proteins and the contents and composition can now be measured (114). Dietary MRPs like pyrraline can be found in the urine originating from blood clearance, and therefore it was speculated that cows excrete AGEs in milk during lactation and thereby expose the suckling newborn. The milk yield of dairy cows has increased significantly over the past decades (115) and thus, a cow needs a ration with a higher energy density and more nitrogen compared to its natural food sources. Thus, the use of processed molasses, soybean meal, and rapeseed meal are increasingly used with the risk of exposure to elevated MRP levels (116). Dietary MRPs, are able to influence the rumen microbiota (117). In addition, also the digestibility of the roughages used to formulate dairy rations, is often low and therefore MRP-containing concentrates that can be absorbed are necessary to ensure dairy productivity. Processed milk proteins using hydrolysis but also heat-induced glycation, display anti-oxidant and anti-inflammatory activities and thereby enhanced functional properties. Raw beef and pork naturally have a small proportion of protein-bound AGEs, while that is much more in raw chicken breasts. Therefore, there is no strong influence of the protein content on the total amount of protein-bound AGEs in beef. The commercial processing strategy, will thus largely determine the final amount of protein-bound AGEs (118).

### AGE and lameness in dairy cattle

One of the most prominent and serious health and welfare problems in dairy cattle worldwide is lameness (or laminitis), mostly due to injury or inflammatory disease in the hoof (119, 120). The prevalence of lameness of dairy cows ranges from 2 to 55% throughout the world depending on area, and has dramatically increased in herds over the past 20 years (121). Apart from the fact that lameness is considered to be a crucial welfare issue, lameness has also a significant economic impact due to a loss in milk production (122–124), reduction in fertility (125–127), and hence an increased risk of culling (128, 129). Laminitis can be defined as a diffuse aseptic inflammation of the dermis of the claw (*Pododermatitis aseptica diffusa*) and is considered to be an important cause of lameness (130–132). It is well-documented that laminitis-related claw lesions including hemorrhage of the sole and the white line along with sole ulcers, are considered to be the most important causes of lameness in dairy cows (119, 133, 134). Many predisposing factors are associated with the occurrence of laminitis including farm management, housing, genetics, breeding, and nutrition (135, 136). Although nutrition is widely related to the development of laminitis, the mechanisms underlying the characteristics of ration and/or feeding management and how these contribute to laminitis occurrence have not yet been extensively studied.

Also in equines, laminitis can occur and the role of obesity and insulin resistance are well known factors that are closely related to the development of laminitis (137, 138, 139). The role of insulin was experimentally confirmed by inducing laminitis in clinically normal horses by prolonged infusions of insulin and glucose to maintain physiological levels of plasma glucose (140). The presence of dietary AGEs was suggested to be involved in the development of insulin-induced laminitis. Also in cattle, the combination of dietary AGEs and insulin resistance in development of laminitis was suggested (141). It has been shown that insulin resistance is often seen in early lactating cows (142, 143), which may be exacerbated by high intakes of rapidly fermentable carbohydrates or starch (144). Interestingly, high prevalence of laminitis lesions, i.e., hemorrhage of the sole and white line are often observed during early lactation (145–148). It can be argued that the occurrence of laminitis during early lactation may be related to insulin resistance and the formation of AGEs.

Advanced glycation end products are derived from glucose through intermediates such as glyoxal, 3-deoxyglucosone and methylglyoxal (149, 150) and it has been postulated that methylglyoxal is a major source of intracellular and plasma AGEs (151). It has been shown that under *in vitro* conditions, the enzymes glyoxalase I and II act in concert to convert methylglyoxal into D-lactate, thereby, preventing the formation of AGEs. In bovines and equines suffering from a systemic acidosis induced by the feeding of high amounts of fermentable carbohydrates and subsequent acidosis of either the rumen or cecum, bovine plasma levels of D- lactate may increase up to 25 mMol/L (152, 153). Unfortunately, the end product D-lactate exerts a negative feedback on the activity of glyoxalase I but this notion may be of interest in relation to the development of laminitis. Apart from the potential that AGEs may be derived from methylglyoxal that originate from the animals intermediary metabolism, methylglyoxal can also be formed during the anaerobic fermentation of rapidly fermentable carbohydrates (154, 155). It was already mentioned that methylglyoxal is converted to D-lactate under physiological conditions. Therefore, it can be speculated that under practical feeding conditions of cows and horses, the fermentation of rapidly fermentable carbohydrates results in the accumulation of both D-lactate and methylglyoxal. Methylglyoxal is toxic to cells (156), which ultimately results in the lysis of bacteria (155) and the subsequent release of lipopolysaccharides, which are implicated in the etiology of laminitis in both bovines (132) and equines (157, 158). Alternatively, methylglyoxal may be absorbed by the rumen epithelium and across the epithelium of the gastro-intestinal tract of bovine and equine and subsequently triggers the formation of AGEs.

## Discussion

Thermal processing of food alters the chemical and biological characteristics of the food components. An example of biochemical changes as a result of heating is the creation of MRPs and AGEs. The MR modified proteins are created in presence of sugars and heat. These molecules are present in various forms in a heterogeneous mixture that justifies their diverse bioactivities.

Feed components and formulation, but also feed processing determine intestinal health and disease resistance. Protein feeds that contain MRP and AGE can cause expansion of intestinal microbiota and together with potentially gut barrier damaging compounds can compromise epithelial barrier function and cause immune stimulation resulting in lower growth, performance and ultimately, in development of disease.

Animals reared and kept in industrial systems are subjected to immunological stress, that together with the pathogen load, the husbandry environment, the feed composition and regimen, and the installed vaccination program determine the immune status. As a consequence, inflammation can occur associated with the release of pro-inflammatory cytokines, the mobilization of nutritional reserves, suppressed nutrient absorption in the gut, and body fluid loss like diuresis and diarrhea. Therefore, inflammation will come at a significant nutrient cost. By activation of the adaptive immune response, specific antibodies will be produced that will consume a relatively small nutrient cost. Thus, dietary immunomodulators and/or vaccines that enhance immune responsiveness and minimize immunological stress will positively affect health, growth, and performance.

The dietary glycated proteins (dAGEs) have shown to have immunomodulatory properties (159, 160). There is substantial evidence to support the association of these glycated proteins with several chronic disorders, which are principally caused due to the accumulation of AGE-modified proteins in cells and tissues (161–164). A probable mechanism for such an immune-stimulatory effect could be the interaction between the AGEs and the antigen presenting cells including macrophages and DCs (165, 166). The pattern recognition receptors present on the cell membrane of these cells recognize the modified proteins, form complexes, and initiate internalization. As a result, activation of the NF-kB transcription factor occurs, leading to the production of pro-inflammatory cytokines and also induction of oxidative stress. Additionally, since macrophages and DCs are APCs, peptides derived from the processed antigens will be presented to CD4+ T-cells on their MHC-II molecules. The combination of presented antigens and secreted cytokine will activate the T-cells and induce cellular responses leading to a chronic inflammatory state if the stimulation persists or the inhibitory mechanisms are inefficient for resolving the homeostasis.

Due to the large diversity of AGEs that are formed during Maillard reactions, various cellular receptors were shown to have binding affinity for these AGEs. Among these receptors, RAGE is the most referred and studied one and sRAGE is the soluble variant of this receptor (167–169). The transmembrane and intracellular signaling domains, which are present in RAGE but not sRAGE, are crucial for transducing the signal to the nucleus and activation of NF-kB (170). However, as sRAGE still carries the ligand-binding domain (V domain), it has a similar binding affinity to AGE-modified proteins as RAGE itself (171). *In vivo*, sRAGE plays a decoy role and binds to circulating AGEs, thereby regulating the interaction between membrane-associated RAGE and the AGEs (110). This interaction between plasma sRAGE and AGEs decreases the risk of undesirable inflammatory response since unlike RAGE, these complexes are assumed to be degraded (172). Indeed, lower plasma levels of sRAGE were reported in patients with chronic inflammation (173). Likewise RAGE, Galectin-3 interacts with the AGEs and contributes to subsequent cell signaling and also uptake of these modified proteins (174). Since this receptor lacks the transmembrane domain, Galectin-3 links to other members of AGE-R complex namely AGE/R1/OST-48 and AGE-R/80K-H. The available CRD on Galectin-3 has an affinity for lactose (175), which may explain the potential interaction with food or feed-derived AGEs. Activation of Galectin-3 leads to an alteration in biological processes including the immune responses and inflammation (176, 177). Furthermore, SR-AI and SR-BIII (CD36) are two members of the macrophage scavenger receptor family that were shown to bind to the glycated proteins (178, 179). These two receptors basically facilitate the endocytosis of the AGEs and are abundantly present on phagocytes (180). The cellular responses following the interaction of these receptors and the AGEs are involved in multiple functions including immune (allergic or/and inflammatory) response (181).

As mentioned before, RAGE is the most recognized receptor for AGEs and the majority of the available studies have focused on this receptor. The structure of the protein is important for driving its biological activities and any modification in this assembly leads to an altered function. As a result of the heat, the hydrogen bonds and polar hydrophobic interactions in secondary and tertiary structures of the proteins are distorted (109). Therefore, the alpha-helixes and beta-sheets are disrupted and the molecule loses its natural folding and 3-dimensional shape. Furthermore, the denatured proteins undergo crosslinking and agglomeration (182). Two or more denatured molecules covalently attach to each other and form new structures that possibly do not match the characteristics of any of the parent molecules. These new molecules are usually rather bulky with altered conformational epitopes that might be recognized by the cellular receptors. Liu et al. showed the association of RAGE binding with whey protein agglomeration (170). In general, the RAGE receptor binding is higher in heated and glycated proteins as they went through structural alterations and became more potent for receptor binding when compared to the unheated samples (183). Generally, glycated proteins (AGEs) have a higher affinity for binding to sRAGE, CD36, SR-AI, and Galectin-3 (16, 183–185).

## Conclusion

In conclusion, during the heating of foods and feeds, protein denaturation, glycation, and agglomeration can occur. These modifications in protein structures, increased their binding affinity to cellular receptors that are mainly present on antigen-presenting cells: namely sRAGE, CD36, SR-AI, and Galectin-3.Although both heated and glycated proteins show an increased receptor binding capacity, the effect of glycation is generally more prominent when compared to the heated one. Despite the proven relation of the MR to affect the protein quality by impairing the bioavailability of amino acids and minerals, decreasing the digestibility and increasing the immunoreactivity of proteins, more information on the physiological and immunological effects of the consumption of MRPs rich diets by animals is urgently needed to the benefit of the health, welfare, growth, and performance of animals.

## Author contributions

MT and HS wrote the manuscript. HW and WH reviewed the manuscript.

### Conflict of interest statement

The authors declare that the research was conducted in the absence of any commercial or financial relationships that could be construed as a potential conflict of interest. The handling Editor declared a shared affiliation, though no other collaboration, with one of the authors WH.
